# Endovascular treatment of acute M1 occlusions due to underlying intracranial atherosclerotic severe stenosis

**DOI:** 10.1186/s41016-022-00292-2

**Published:** 2022-09-01

**Authors:** Yazhou Yan, Li Du, Xiliang He, Qinghai Huang, Yuan Pan, Tao Xin

**Affiliations:** 1 Stroke Center of 971 Hospital of PLA, Qingdao, 266071 China; 2 Department of Neurosurgery of Changhai Hospital affiliated to the Naval Military Medical University, Shanghai, China

**Keywords:** Endovascular treatment, M1 occlusion, Intracranial severe stenosis, Rescue therapy

## Abstract

**Background:**

Endovascular treatment (EVT) for acute ischemic stroke with an occlusion of the M1 segment due to intracranial atherosclerotic severe stenosis (ICASS) remains challenging. This study aimed to evaluate the safety and efficacy of EVT for ICASS-related M1 acute occlusion.

**Methods:**

We retrospectively reviewed all patients with an ICASS-related M1 acute occlusion who underwent EVT at our institution between January 2015 and December 2020. Clinical presentation, baseline characteristics, angiographic and clinical results, technical feasibility, perioperative complications, and follow-up results were evaluated.

**Results:**

Twenty-two patients with ICASS-related M1 acute occlusion were included. Eight patients (36.4%) received bridging therapy, and the other 14 patients (63.6%) directly underwent EVT. Fifteen patients (68.2%) treated with balloon dilations and stenting as rescue treatment. Six patients (27.3%) received single balloon angioplasty, and 5 of these patients were treated with staged stenting. One case (4.5%) failed recanalization at the first EVT, and successful revascularization was achieved a month later. The mean procedure time was 67.2 ± 20.8 min. Successful revascularization (mTICI ≥ 2b) was achieved in 95.5% (21/22) of patients. Perioperative complications developed in two patients (9.1%) including one hemorrhagic event and one thromboembolic event. Angiographic follow-up was available in 20 patients (90.9%) at an average of 8.6 ± 3.0 months. The degree of stenosis was worse (10–30%) in 6 cases (30%) compared with the initial outcomes. The favorable outcomes (mRS ≤ 2) at 3-month follow-up was achieved in 19 patients (86.4%).

**Conclusions:**

ICASS-related occlusion in the M1 segment often required a rescue therapy including balloon angioplasty with/without stenting, and this treatment strategy was safe and effective. But single balloon angioplasty at the first EVT generally cannot achieve satisfactory results and often needed staged stenting treatment.

## Background

Endovascular treatment (EVT) has become one of the primary therapies for acute ischemic stroke patients with large-vessel occlusion in the anterior circulation after the famous randomized controlled trials conducted in recent years [1–7]. Intracranial atherosclerotic stenosis (ICAS) is one of the most common etiologies of ischemic stroke in the world and is more prevalent in Asian, Hispanic, and Black populations than in Caucasians [8]. However, the optimal treatment for ICAS-related occlusion of intracranial large arteries remains unclear. During endovascular procedures of ICAS-related occlusion, residual stenosis and instant reocclusion of the treated arteries are more frequent and often require rescue therapies including balloon or/and stent angioplasty [9]. In addition, antiplatelet drugs are frequently used after the deployment of stent, which increases the risk of hemorrhagic events. Hence, we conducted this retrospective study to evaluate the safety and efficacy of EVT for acute ischemic stroke patients with an occlusion of the middle cerebral artery (MCA) M1 segment due to an underlying intracranial atherosclerotic severe stenosis (ICASS) in our center.

## Methods

### Patients

We retrospectively reviewed all stroke patients with ICASS-related M1 occlusion who underwent EVT at our institution between January 2015 and December 2020. The inclusion criteria were as follows: (1) age ≥ 18 years, (2) isolated M1 occlusion confirmed by CTA or MRA, (3) acute symptom onset with a neurologic deficit in accordance with the occluded MCA territory, and (4) patients who underwent EVT and confirmed as ICASS-related occlusion. The exclusion criteria were as follows: (1) multiple vessel occlusions or tandem occlusions; (2) another stroke etiology such as dissection, moyamoya disease, or vasculitis; and (3) data (baseline or procedural or follow-up) were missing. Two experienced interventional neurologists assessed the surgical notes, medical charts, and radiologic images of the patients. Moreover, patients’ demographics (age and gender), risk factors (diabetes, hypertension, hyperlipidemia, atrial fibrillation, smoking, drinking, and previous stroke), clinical and imaging characteristics, perioperative complications, and immediate postoperative results were evaluated. An ICASS-related occlusion was defined as a degree of fixed stenosis > 70% after thrombectomy in the occlusion site, not sensitive to vasoactive substances, with impairment of perfusion of the dependent territory. This retrospective study was approved by the Institutional Review Board of 971 Hospital of PLA (LL20220608002).

### Procedure details

For the patients with proven occlusion of the M1 segment within the time window of intravenous thrombolysis and without contraindications, they received standard care which started with intravenous alteplase (0.9 mg/kg) according to the guidelines, before undergoing EVT. Patients over 4.5 h were directly subjected to EVT. The physician and anesthetists decided the choice of anesthesia (local or general) on the patient’s consciousness status. Patients who treated with bridging EVT protocol received no heparin, while the others were administered with a low dose of heparinization. The treating physician selected the type of EVT procedure according to the angiographic result. An 8-F or 9-F guiding catheter was introduced into the internal carotid artery to provide stable support. The direct aspiration thrombectomy (Penumbra, Alameda, CA, USA), stent retrievers (Solitaire, Medtronic, Irvine, CA, USA; Trevo, Stryker Neurovascular, Fremont, CA, USA), or combinations were routinely performed. For patients with a degree of stenosis > 70% after thrombectomy, a vasodilator was injected through the guiding catheter for continuous irrigation, and repeat angiography was performed to exclude potential dissection or vasospasm after the first recanalization. If the stenosis persisted, an underlying ICASS was identified.

Once ICASS-related occlusion was identified, after the cone-beam CT showed no hemorrhage, a low dosage of tirofiban (glycoprotein IIb/IIIa inhibitor) was injected, and a rescue therapy strategy was applied. Then, intracranial angioplasty was performed with a Gateway balloon (Boston Scientific, Natick, MA, USA). The diameter of the balloon was adjusted to 80% of the normal vessel diameter, and the shortest length that would cover the entire lesion was chosen to minimize potential damage to lenticulostriate arteries in adjacent unaffected segments [10]. The repeat angiography was performed, and when stenosis persisted or reocclusion occurred, permanent stent (Solitaire; Enterprise, Codman, Raynham, MA, USA; or Neuroform, Stryker Neurovascular, Fremont, CA, USA) was deployed. All patients underwent CT scans immediately after procedure, and 24 h after, to detect any intracranial hemorrhage. When the patient’s neurological status deteriorated, a CT scan was immediately performed. If no intracranial hemorrhage was detected, tirofiban was continuously used in the patients with ICASS-related occlusion. Meanwhile, dual antiplatelet therapy with clopidogrel and aspirin (300 mg each) was administered and overlapped with tirofiban infusion 6 h before the cessation of tirofiban for these patients with stent deployment. And then, these patients were treated for 3 months with aspirin (100 mg/day) and clopidogrel (75 mg/day), followed by lifelong 100mg aspirin.

### Clinical assessment and follow-up

**Fig. 1:**
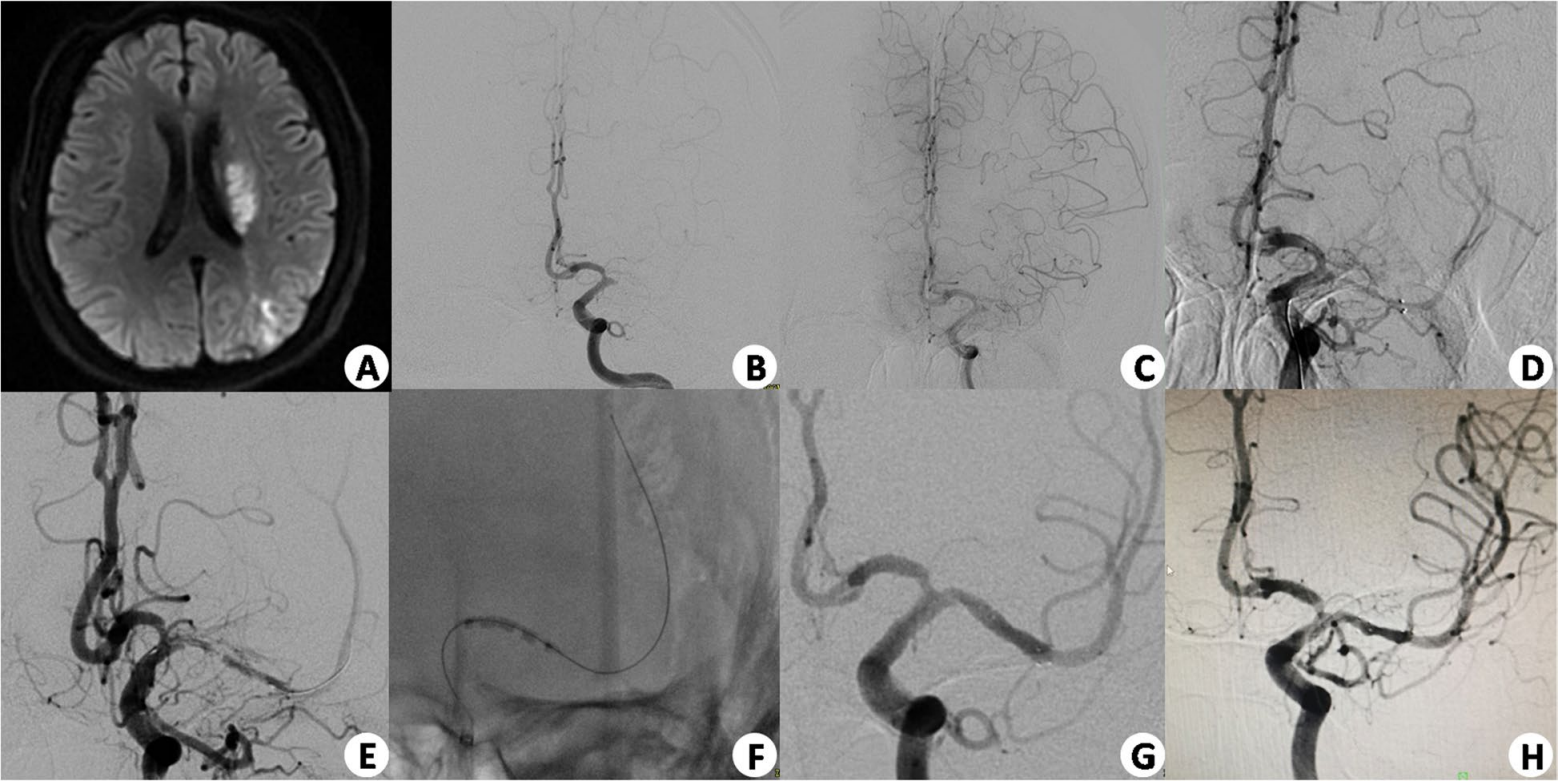
A 29-year-old male patient with acute ischemic stroke receiving bridging therapy, NIHSS 10. **A** Preoperative MRI showed an acute infarction in the left cerebral hemisphere. **B**, **C**, and **D** DSA showed left M1 occlusion with good collaterals, and this patient was treated with endovascular thrombectomy. **E** Repeat angiography showed an intracranial atherosclerotic severe stenosis in the lesion. **F** and **G** This patient received a rescue therapy including balloon angioplasty (Gateway 2.5 × 15 mm) with stenting (Neuroform 3.5 × 15 mm), and the outcome was mTICI 3. **H** A 6-month follow-up angiogram showed good stent apposition to the parent artery wall, and the degree of stenosis was worse compared with the initial outcomes after procedure

Successful reperfusion was defined as modified Thrombolysis in Cerebral Infarction (mTICI) score of 2b–3 [11]. The National Institutes of Health Stroke Scale (NIHSS) was assessed by experienced neurosurgeons. Clinical outcome was assessed using the modified Rankin Scale (mRS) at 3-month follow-up at the clinic or by phone interview. The first angiographic follow-up was generally performed at 6 months using DSA and then MRA or DSA yearly thereafter. For the patients, underlying ICASS-related occlusion treated with intracranial angioplasty alone and the stenosis has improved significantly (degree of stenosis ≤ 50%); once patients were clinically and neurologically recovered from the acute phase after stroke (2 weeks), DSA was performed. If the stenotic segment was severe restenosis (degree of stenosis > 70%), stent deployment was scheduled Fig. 1.

## Results

### Baseline characteristics

A total of 109 acute stroke patients with an isolated M1 occlusion underwent EVT at our institution between January 2015 and December 2020. There were 22 (20.2%) patients with ICASS-related M1 occlusion included. The patients consisted of 13 men and 9 women with a mean age of 57.8 ± 16.7 years (range 29–86 years). Patient risk factors included diabetes (27.3%), hypertension (50.0%), hyperlipidemia (59.1%), atrial fibrillation (4.5%), smoking (36.3%), drinking (18.2%), and previous stroke (27.3%). Twelve patients (54.5%) had ICASS-related occlusion in the left MCA. And there were 3 cases (13.6%), 15 cases (68.2%), and 4 cases (18.2%) in the proximal, middle, and distal M1 segment, respectively. Patients presented with a mean NIHSS score of 11.2 ± 7.8 (range 4–29) at admission. Data are summarized in Table 1.

**Table 1:** Baseline characteristics of patients

Variable	Values^a^
Age (yr)	57.8 ± 16.7
Sex	
Male	13 (59.1%)
Female	9 (40.9%)
NIHSS	11.2 ± 7.8
Risk factors	
Diabetes mellitus	6 (27.3%)
Hypertension	11 (50.0%)
Hyperlipidemia	13 (59.1%)
Atrial fibrillation	1 (4.5%)
Previous stroke	6 (27.3%)
Smoking	8 (36.3%)
Drinking	4 (18.2%)
Location	
L	12 (54.5%)
R	10 (45.5%)
Intravenous alteplase	8 (36.4%)
Onset to puncture time (min)	268.3 ± 91.4

^a^Values are mean ± SD or number of patients (percentage). *NIHSS*, National Institutes of Health Stroke Scale; *L*, left; *R*, right

### Procedural details

Eight patients (36.4%) received bridging therapy, and the other 14 patients (63.6%) directly underwent EVT. Fifteen patients (68.2%) were treated with noncompliant balloon dilations and stent implantations as rescue treatment. Six patients (27.3%) received single balloon angioplasty, and 5 of these patients were treated with staged stenting within 1.0 ± 0.6 months (range 0.5–2.0 months). One case (4.5%) failed recanalization at the first EVT. And successful revascularization was achieved a month later, and balloon dilations and stenting were treated in this patient. The mean procedure time was 67.2 ± 20.8 min (range 40–100 min). Successful revascularization (mTICI ≥ 2b) was achieved in 95.5% (21/22) of patients, including 2 in 2b and 19 in 3. Data are summarized in Table 2.

**Table 2:** Procedural details and outcomes

Variable	Values^a^
General anesthesia	8 (36.4%)
Procedure time (min)	67.2 ± 20.8
Treatment strategy	
Balloon angioplasty and stenting	15 (68.2%)
Balloon angioplasty alone	6 (27.3%)
Successful revascularization	21 (95.5%)
Postoperative mTICI	
0	1 (4.5%)
1	—
2a	—
2b	2 (9.1%)
3	19 (86.4%)
Complications	
Hemorrhagic event	1 (4.5%)
Thromboembolic event	1 (4.5%)
Angiographic follow-up (months)	8.6 ± 3.0
mRS score at 3 months	
0	7 (31.8%)
1	6 (27.3%)
2	6 (27.3%)
3	2 (9.1%)
4	—
5	—
6	1 (4.5%)

^a^Values are mean ± SD or number of patients (percentage). *mTICI*, modified Thrombolysis in Cerebral Infarction; *mRS*, modified Rankin Scale

### Complications and outcomes

Perioperative complications developed in two patients (9.1%) including one hemorrhagic event and one thromboembolic event. In one patient, CT scan 24 h after procedure indicated a small amount of intracerebral hemorrhage in the basal ganglia. After conservative treatment, this patient recovered well and got a mRS score of 1 at the time of his 3-month follow-up visit. Another patient received single balloon angioplasty at the first EVT and deteriorated clinically the day after surgery. The CT images did not show any signs of intracranial hemorrhage, and MRA showed the reocclusion of the M1 segment which eventually caused the death of this patient. The favorable outcomes (mRS ≤ 2) at 3-month follow-up were achieved in 19 patients (86.4%). Angiographic follow-up was available in 20 patients (90.9%). The mean follow-up time of these patients was 8.6 ± 3.0 months (range 5 to 15 months). The degree of stenosis was worse (10–30%) in 6 cases (30%) compared with the outcomes immediately after procedure. No hemorrhagic or thromboembolic complications occurred during the follow-up. Data are summarized in Table 2.

## Discussion

The ICASS-related M1 occlusion might suffer relatively low baseline NIHSS which could lead to a missed diagnosis, and during EVT for this lesion, residual stenosis and instant reocclusion of the treated arteries might be frequent. All of these features increased the difficulty of treatment. This single-center study retrospectively analyzed the safety and efficacy of EVT for acute ischemic stroke patients with an occlusion of the M1 segment due to an underlying ICASS, showing that ICASS-related occlusion in the M1 segment often required a rescue therapy including balloon angioplasty and stenting, and single balloon angioplasty at the first EVT generally cannot achieve satisfactory results and often needed staged stenting treatment. Moreover, antiplatelet therapy for stenting in this study including the low-dosage tirofiban (5 μg/kg intravenous bolus within 3 min, maintained with 0.075 μg/kg/min) overlapped with dual antiplatelet therapy (aspirin and clopidogrel) did not increase the risk of hemorrhagic or thromboembolic complications.

Intracranial atherosclerosis was more popular in the Eastern than in the Western. Recent studies have suggested that ICAS-related occlusion accounted for about one-third of all ischemic stroke etiologies in Asia [6, 12]. And according to a meta-analysis, more than half of the ICAS-related occlusions were present in the MCA [9]. Mechanical thrombectomy was primarily designed for embolic occlusion rather than ICAS-related occlusion [6]. It may produce initial recanalization quickly. But it also could lead to mechanical disruption of atherosclerotic plaques or endothelial injury which might trigger platelet activation, adhesion, and aggregation and result in thrombosis and reocclusion, especially for the ICASS-related M1 occlusion. Therefore, several previous studies evaluated the safety and efficacy of EVT in patients with acute ICAS-related occlusion and come to discrepant conclusions. Dobrocky et al. [10] reported 10 patients underlying ICAS-related M1 acute occlusion treated with EVT. The rate of reperfusion success was 70%. But the favorable outcome at 90 days was 0, and the mortality rate was 44.4%. Lee et al. [13] reported similar recanalization rate (76.8%) and poor clinical outcomes with the low rate of 3-month functional independence (45.5%) in patients with ICAS. However, Jia et al. [12] performed a prospective study in China about ICAS-related occlusion and showed the high rates of successful recanalization (95.7%) and functional independence at 90 days (63.8%). Yang et al. [14] reported a similar recanalization rate (97.8%) and more favorable outcomes in patients with ICAS. Also, a meta-analysis performed by Li et al. [9] showed the safety and effectiveness of EVT for ICAS-related occlusions. This discrepancy may be associated with different definition of ICAS and occlusion site and inhomogeneous population. In our study, successful revascularization (mTICI ≥ 2b) was achieved in 95.5%, and favorable outcomes at 3 months were achieved in 19 patients (86.4%).

This study presented a relatively high rate of favorable outcomes with EVT for ICASS-related M1 occlusion which might be due to several factors. Firstly, the baseline NIHSS in this study was 11.2 ± 7.8, which was relatively low and lower than the initial NIHSS score (16.0) in the meta-analysis performed by Li et al. [9] about ICASS-related occlusion in the large vessel. And previous studies have shown that initial NIHSS score was an independent predictor of favorable outcomes [13, 15]. The relatively low initial NIHSS may be due to ischemic preconditioning and good collaterals. Collateral circulation could protect the brain parenchyma from ischemic damage when the normal antegrade flow is obstructed. These patients with ICAS had better collaterals than those with other stroke subtypes like embolism, presumably due to that the ICAS required a longer time for the complete occlusion of arteries, which allowed the development of adequate collaterals before the acute stroke [16]. Secondly, patients underlying ICASS had relatively lower clot burden compared with the embolism-related occlusion. We could achieve recanalization as soon as possible, which would reduce the time of brain tissue ischemia. Also, a light clot burden could lead to a lower risk of distal embolization during EVT, which could result in higher rate of successful recanalization. Finally, it is said that the longer procedure time which reflected the procedure complexity could lead to worse outcomes [10, 13]. In this study, due to techniques and catheters for thrombectomy evolving dramatically and our experience accumulation, the procedure time was 67.2 ± 20.8 min which was shorter than that in previous studies [10, 17]. In this study, if there was severe stenosis with insufficient reperfusion or reocclusion occurred during the EVT, a balloon angioplasty with/without stent would be performed as soon as possible. And repeated thrombectomy would be avoided, which could aggravate the disruption of plaque and result in reocclusion of the lesion. Moreover, good collaterals in the ICAS-related occlusion might sustain the blood supply of the walls of the occlusion artery, which could reduce the risk of blood leakage [12] and could reduce the damage caused by hyperperfusion after the recanalization, so that intracranial hemorrhage after procedure would reduce.

Although the ICASS-related M1 occlusion had lower clot burden compared with the embolism-related occlusion and could achieve recanalization more easily, but the stenotic vessel segment always had a vulnerable plaque. After endovascular thrombectomy, the plaque was further disrupted, and local thrombosis was formatted which leaded to early reocclusion [18], so that additional rescue treatments such as balloon angioplasty and stenting were often required. It is said that intracranial stenting has been shown to be more effective than angioplasty alone for postoperative residual stenosis or late restenosis in cases of ICAS-related occlusion [19]. In this study, 6 patients were treated with balloon angioplasty alone at first EVT and needed staged stenting within 1.0 ± 0.6 months after procedure. Therefore, antithrombotic treatment was generally recommended to prevent acute thrombosis and reocclusion in cases of stent placement. But, optimal dosages of perioperative antiplatelet therapy have not yet been established, and hemorrhagic risk cannot be ignored. Previous studies [10, 20] have shown that mono and dual antiplatelet therapies with aspirin and clopidogrel may not be sufficient in the acute setting, and they thought glycoprotein IIb/IIIa inhibitors should be considered. Tirofiban, a platelet glycoprotein IIb/IIIa antagonist, has been commonly used in acute coronary syndrome. Recently, it has been used in patients with underlying ICAS after mechanical thrombectomy [14]. In this study, a low-dosage tirofiban (5 μg/kg intravenous bolus within 3 min, maintained with 0.075 μg/kg/min) was administered to the patients if the intraoperative CT showed no hemorrhage and overlapped with dual antiplatelet therapies. There were 2 perioperative complications (9.1%) including one hemorrhagic event and one thromboembolic event occurred in this study.

There were several limitations of our study, including its retrospective design, patient selection bias, and the limited sample size from a single center. In addition, due to the different prevalence of ICAS worldwide, the treatment experience in Asia was more extensive than that in Europe and North America. In the future, a study with multicenter larger sample size and longer follow-up are needed to perform.

## Conclusions

In this study, we found that ICASS-related occlusion in the M1 segment often required a rescue therapy including balloon angioplasty with/without stenting, and this treatment strategy was safe and effective. But single balloon angioplasty at the first EVT generally cannot achieve satisfactory results and often needed staged stenting. Moreover, antiplatelet therapy for stenting in this study including the low-dosage tirofiban overlapped with dual antiplatelet therapy (aspirin and clopidogrel) did not increase the risk of hemorrhagic or thromboembolic complications.

## Data Availability

Please contact author for data requests.

## Abbreviations

EVT: Endovascular treatment
ICASS: Intracranial atherosclerotic severe stenosis
MCA: Middle cerebral artery
mTICI: Modified thrombolysis in cerebral infarction
mRS: Modified Rankin Scale
NIHSS: National Institutes of Health Stroke Scale
